# Correction: Carotenuto et al. *Xenopus laevis* (Daudin, 1802) as a Model Organism for Bioscience: A Historic Review and Perspective. *Biology* 2023, *12*, 890

**DOI:** 10.3390/biology14111587

**Published:** 2025-11-13

**Authors:** Rosa Carotenuto, Maria Michela Pallotta, Margherita Tussellino, Chiara Fogliano

**Affiliations:** 1 Department of Biology, University of Naples Federico II, 80126 Naples, Italy; tussemar@libero.it; 2 Institute for Biomedical Technologies, National Research Council, 56124 Pisa, Italy; mariamichela.pallotta@irgb.cnr.it

## Figure Legend

In the original publication [1], there was a mistake in the caption of Figures 1 and 2. In the originally submitted manuscript, the illustrations of *Xenopus* developmental stages included in Figures 1 and 2 were reproduced from Xenbase resources without appropriate attribution. These illustrations were created by © Natalya Zahn (2022) as part of the publication “Normal Table of *Xenopus* Development: A New Graphical Resource” [15] and are publicly available via Xenbase. The correct captions appear below. 

**Figure 1.** Scheme of Frog Embryo Teratogenesis Assay—*Xenopus* (FETAX), employed to measure the activity of pollutants during embryo development. *Xenopus* illustrations © Natalya Zahn (2022) and sourced from Xenbase (www.xenbase.org RRID: SCR_0032803; Accessed on 12 January 2023) [15].

**Figure 2.** Life cycle of *X. laevis* staged according to Nieuwkoop and Faber (1956) [6]. Through in vitro and in vivo fertilization, it is possible to follow all stages of early embryonic development. *Xenopus* illustrations © Natalya Zahn (2022) and sourced from Xenbase (www.xenbase.org RRID: SCR_0032803; Accessed on 12 January 2023) [15].

## Reference

The newly added reference appears below:

15. Zahn, N.; James-Zorn, C.; Ponferrada, V.G.; Adams, D.S.; Grzymkowski, J.; Buchholz, D.R.; Nascone-Yoder, N.M.; Horb, M.; Moody, S.A.; Vize, P.D.; et al. Normal Table of *Xenopus* development: A new graphical resource. *Development* **2022**, *149*, dev200356. https://doi.org/10.1242/dev.200356.

With this correction, the order of some references has been adjusted accordingly. The authors state that the scientific conclusions are unaffected. This correction was approved by the Academic Editor. The original publication has also been updated.
